# Preparation, Characterization and Evaluation of Quetiapine Fumarate Solid Lipid Nanoparticles to Improve the Oral Bioavailability

**DOI:** 10.1155/2013/265741

**Published:** 2013-06-03

**Authors:** Arjun Narala, Kishan Veerabrahma

**Affiliations:** Department of Pharmaceutics, University College of Pharmaceutical Sciences, Kakatiya University, Warangal, Andhra Pradesh 506009, India

## Abstract

Quetiapine fumarate is an antipsychotic drug with poor oral bioavailability (9%) due to first-pass metabolism. Present work is an attempt to improve oral bioavailability of quetiapine fumarate by incorporating in solid lipid nanoparticles (SLN). Six quetiapine fumarate SLN formulations were developed using three different lipids by hot homogenisation followed by ultrasonication. The drug excipient compatibility was studied by differential scanning calorimetry (DSC). Stable quetiapine fumarate SLNs having a mean particle size of 200–250 nm with entrapment efficiency varying in between 80% and 92% were developed. The physical stability of optimized formulation F3 was checked at room temperature for 2 months. Comparative bioavailability studies were conducted in male Wistar rats after oral administration of quetiapine fumarate suspension and SLN formulation. The relative bioavailability of quetiapine fumarate from optimized SLN preparation was increased by 3.71 times when compared with the reference quetiapine fumarate suspension. The obtained results are indicative of SLNs as potential lipid carriers for improving the bioavailability of quetiapine fumarate by minimizing first-pass metabolism.

## 1. Introduction 

Quetiapine fumarate is an antipsychotic drug with plasma half life of 6 h and poor oral bioavailability (9%) due to extensive first-pass metabolism [1]. Possible methods to avoid first-pass metabolism include transdermal, buccal, rectal, and parenteral routes of administration. Oral route is the most commonly used and preferred route for the delivery of drugs, although several factors like pH of GIT, residence time, and solubility can affect drug absorption or availability by this route. Lymphatic delivery is an alternative choice to avoid first-pass metabolism in oral drug delivery. Enhanced lymphatic transport of drugs reduces the hepatic first-pass metabolism and improves oral bioavailability, because intestinal lymph vessels drain directly into thoracic duct, further in to the venous blood, thus bypassing the portal circulation [2, 3]. The main function of the lymphatic system is to facilitate absorption of long-chain fatty acids via chylomicron formation. Two different lipid-based approaches are known to enhance the lymphatic transport, which include construction of a highly lipophilic prodrug and incorporation of drug in a lipid carrier [4]. 

Solid lipid nanoparticles (SLNs) are an alternative nanoparticulate carrier system to polymeric nanoparticles, liposomes, and o/w emulsions [5–8]. Aqueous SLN dispersions are composed of lipid which is solid at both body and room temperatures, being stabilized by a suitable surfactant. SLNs possess distinct advantages compared to other carriers, for example, polymeric nanoparticles; lipids included in topical and oral drug delivery can be used as matrix material, including the long list of different surfactants/stabilizers employed in these traditional formulations. Thus, there is no problem with the regulatory accepted status of excipients [8]. Lipid nanoparticles were studied for percutaneous drug delivery [9, 10]. SLNs also enjoy more advantages over other colloidal delivery systems with regard to biocompatibility, scaleup, and also the release of drugs from SLNs which can be modulated in order to optimize their performance [11]. These features make lipid nanoparticles an interesting carrier system for optimized oral delivery of drugs.

There are very few reports in literature describing the use of SLNs for bypassing first-pass metabolism. When all-trans-retinoic acid was loaded into SLNs, the oral bioavailability in rats was increased four- to five-fold compared with that of suspension [12]. The oral bioavailability of cryptotanshinone was increased by incorporating into SLNs [13]. The pharmacokinetics and tissue distribution aspects of clozapine loaded solid lipid nanoparticles after intraduodenal administration were studied [14].

In the present study, the quetiapine fumarate loaded SLNs were prepared using glyceryl trimyristate, glyceryl tristearate, and glyceryl monostearate as lipids by hot homogenization followed by ultrasonication method. The prepared SLNs were characterized, and an optimized SLN formulation was used to assess the oral bioavailability improvement of quetiapine fumarate. 

## 2. Materials and Methods

### 2.1. Materials

Quetiapine fumarate was obtained as a gift sample from Aurobindo Labs, Hyderabad. Dynasan 114 (glyceryl trimyristate), Dynasan 118 (glyceryl tristearate) and Imwitor 900P (glyceryl monostearate) (Sasol, Witten, Germany), egg lecithin (Lipoid, Germany), Poloxamer-188 (Himedia, Mumbai), chloroform (Qualigens, India), methanol (Rankem, India), dialysis membrane (HiMedia, Mumbai) were purchased from the local market. All the other reagents used were of analytical grade.

### 2.2. Preparation of Quetiapine Fumarate Loaded Solid Lipid Nanoparticles

Quetiapine fumarate loaded SLNs were prepared by hot homogenization followed by the ultrasonication [7]. The composition of various formulations is shown in Table 1. Quetiapine fumarate, solid lipid, and emulsifier (egg lecithin) were dissolved in 10 mL of a mixture of methanol and chloroform (1 : 1). Organic solvents were completely removed using a rotary flash evaporator. The embedded lipid layer was molten by heating to 5°C above the melting point of the lipid. An aqueous phase was prepared by dissolving the stabilizer (poloxamer 188) in distilled water (1.5% w/v) and heated to the same temperature of the oil phase. The hot aqueous phase was added to the oil phase, and homogenization was performed (at 12000 rpm) using a homogenizer (DIAX 900 Heidolph, Germany) for 5 min. The coarse oil in water emulsion obtained was sonicated using a probe (12T) sonicator (Vibracell Sonics, USA) for 20 min. Quetiapine fumarate loaded SLNs were finally obtained by allowing the hot nanoemulsion to cool to room temperature. In this study, quetiapine fumarate loaded SLNs were prepared using three lipids, each at two different concentrations. 

### 2.3. Characterization of Solid Lipid Nanoparticles

#### 2.3.1. Drug-Excipient Compatibility Studies by Differential Scanning Calorimeter (DSC)

DSC scan was performed by Mettler-Toledo DSC 821e (Columbus, OH, USA) instrument. DSC scans were recorded for the entire drug and lipid combinations at a heating rate of 10°C/min in temperature range of 50–250°C. 

#### 2.3.2. Measurement of Particle Size, Poly Dispersity Index (PDI), and Zeta Potential (ZP) of SLN

The size, PDI, and ZP of quetiapine fumarate SLNs were measured using a Malvern Zetasizer (Nano ZS90, UK). About 100 *μ*L of the prepared SLN dispersion was diluted to 5 mL with double distilled water and analyzed with zetasizer.

#### 2.3.3. Determination of Entrapment Efficiency (EE)

Entrapment efficiency was determined by measuring the concentration of free drug (unentrapped) in aqueous medium as reported [14]. The aqueous medium was separated by ultrafiltration using centrisart tubes (Sartorius, USA) which consisted of filter membrane (M.Wt. cut-off 20,000 Da) at the base of the sample recovery chamber. About 2.5 mL of the formulation was kept in the outer chamber, and sample recovery chamber was placed over the sample and centrifuged at 4000 rpm for 15 min. The SLN along with the encapsulated drug remained in the outer chamber and aqueous phase moved into the sample recovery chamber through filter membrane. The amount of quetiapine fumarate in the aqueous phase was estimated by HPLC.

#### 2.3.4. Determination of Total Drug Content

About 100 *μ*L of the formulation was dissolved in 0.9 mL of chloroform and methanol mixture (1 : 1), and then further dilutions were made with mobile phase. The diluted samples were estimated by HPLC for the amount of quetiapine fumarate present [14].

#### 2.3.5. *In Vitro* Drug Release Studies

*In vitro* release studies were performed using dialysis method. Dialysis membrane (Himedia, India) having pore size 2.4 nm and molecular weight cut-off between 12,000–14,000 was used for the release studies. Dialysis membrane was soaked overnight in double distilled water prior to the release studies. Hydrochloric acid (0.1 N) and phosphate buffer pH 6.8 were used as release media. The experimental unit had donor and receptor compartments. Donor compartment consisted of a boiling tube which was cut open at one end and tied with dialysis membrane at the other end into which 1 mL of SLN dispersion was taken for release study. Receptor compartment consisted of a 250 mL beaker which was filled with 100 mL release medium, and the temperature was maintained at 37 ± 0.5°C. At 0.25-, 0.5-, 1-, 2-, 3-, 4-, 6-, 8-, 10-, 12-, and 24-hour time points, 1 mL samples were withdrawn from receiver compartment and replenished with the same volume of release medium. The collected samples were suitably diluted and analyzed by UV-visible spectrophotometer (SL-150, ELICO, India) at 209 nm [15].

#### 2.3.6. Physical Stability Studies

Quetiapine fumarate loaded solid lipid nanoparticles (optimized formulation F3) were stored at room temperature (25°C/60 ± 5% RH) and refrigerated temperature (4°C) for 60 days, and average size, zeta potential, poly dispersity index and EE were determined in triplicate.

### 2.4. Bioavailability Study 

#### 2.4.1. Study Design and Sampling Schedule

A single dose bioavailability study was designed in male Wistar rats under fasting conditions. The oral bioavailabilities of the optimized SLN formulation (F3) and suspension (F7) were estimated by conducting bioavailability studies in male Wistar rats with oral dose of 10 mg/kg body weight. All experimental procedures were reviewed and approved by the institutional animal ethical committee of University College of Pharmaceutical Sciences, Kakatiya University (Warangal, India). Male Wistar rats weighing 200–250 g were taken for study (6 animals per group). Blood samples were withdrawn by retro-orbital venous plexus puncture at 0.5, 1, 1.5, 2, 3, 4, 6, 8, 10, 12, and 24 h after dose. About 1.5 mL of blood samples were withdrawn in eppendorf tubes and centrifuged at 3000 rpm for 30 min. The plasma was transferred to another eppendorf tube and stored at −20°C until analysis.

#### 2.4.2. Serum Sample Processing and High-Performance Liquid Chromatographic (HPLC) Analysis of Quetiapine Fumarate

To 100 *μ*L of serum, 10 *μ*g/mL of felodipine (internal standard) was added, and then alkalinization was achieved by the addition of 0.1 mL of NaOH (0.1 M), and the tubes were shaken for 1 min. Then about 5 mL of ether was added and vortexed for 5 min. After vortex mixing for 5 min, the mixtures were centrifuged at 3000 rpm for 6 min at room temperature. Of the upper layer, 4 mL was carefully aspirated, and the remainder was extracted once again with 5 mL of ether. Of the upper layer, 4 mL was collected together with the former. The ether was evaporated at room temperature. The residue was reconstituted with 100 *μ*L of methanol, and reconstituted samples were analyzed using HPLC [16].

#### 2.4.3. Chromatographic Conditions

 Mobile phase: acetonitrile: 0.02 M phosphate buffer pH 5.5 (65 : 35),
 flow rate: 1 mL/min,
 column: Lichrospher C-18 (250 mm × 4.6 mm i.d., 5 *μ*m particle size),
 injection volume: 20 *μ*L,
 UV detection: 254 nm,
 retention time: 5.2 min.

#### 2.4.4. Calculation of Pharmacokinetic Parameters

The concentrations of quetiapine fumarate in rat serum samples were obtained from the calibration curve prepared. The pharmacokinetic parameters *C*_max_, *T*_max_, AUC_0–24_, and *t*_1/2_ were calculated by Kinetica software.

The relative bioavailability (BA) of quetiapine fumarate SLNs to the oral suspension was calculated as follows:

(1) $$\begin{array}{c} \text{Relative}\;\text{BA}=\left(\frac{{\text{AUC}}_{\text{SLN}}\times {\text{Dose}}_{\text{Suspension}}}{{\text{AUC}}_{\text{Suspension}}\times {\text{Dose}}_{\text{SLN}}}\right). \end{array}$$

## 3. Results and Discussion

In the present study, quetiapine fumarate loaded SLNs were prepared by hot homogenisation followed by ultrasonication method using three lipids, each at two different concentrations. The selection and utility of lipids and method of preparation were based on the earlier reports. Dynasan-114, Dynasan-118, and Imwitor-900P were known to produce SLNs. Due to the reports and previous observations, these lipids were tried in our studies as excipients. Egg lecithin and poloxamer were known as surfactants to get the dispersions of SLNs. At first instance, hot homogenization followed by ultrasonication method was tried, which yielded better SLNs. Inspite of availability of other methods of preparation, we used this method only as this resulted in consistent production of smaller size nanoparticles (<250 nm) with narrow size distribution and good entrapment efficiency.

### 3.1. Drug-Excipient Compatibility Studies by DSC

**Figure 1:**
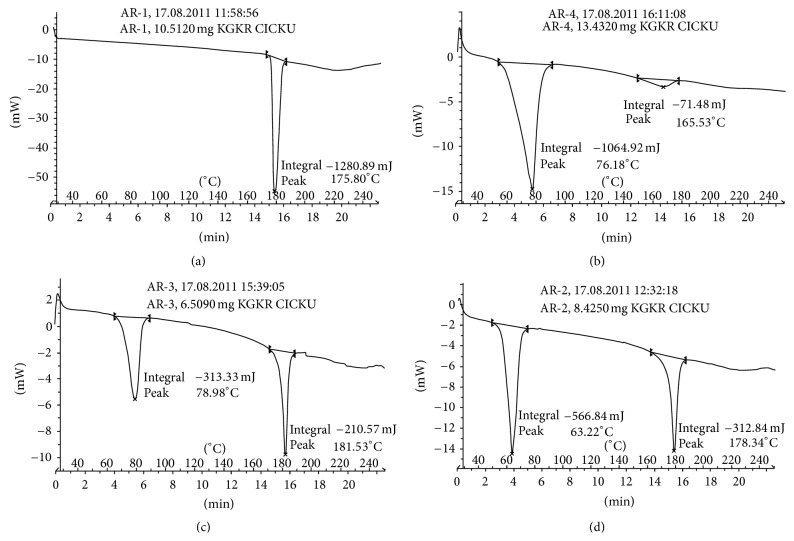
DSC thermograms of (a) pure drug, (b) physical mixture of drug and Imwitor-900P, (c) physical mixture of drug and Dynasan-118 and (d) physical mixture of drug and Dynasan-114.

DSC thermograms of pure drug and physical mixtures of drug and different lipids are shown in Figure 1. The pure drug, quetiapine fumarate, showed a sharp endothermic peak at 175.80°C (Figure 1(a)). Physical mixture of drug and Imwitor-900P showed broad and sharp endothermic peaks at 165.53°C and 76.18°C (Figure 1(b)), respectively. Physical mixture of drug and Dynasan-118 showed sharp endothermic peaks at 181.53°C and 78.98°C (Figure 1(c)), respectively. Physical mixture of drug and Dynasan-114 showed sharp endothermic peaks at 178.34°C and 63.22°C (Figure 1(d)), respectively. 

Drug-related peaks are seen at right side of thermograms and lipid-related peaks are on left side of the Figure 1. In all the cases, melting endotherm of drug was well preserved with slight changes in terms of broadening or shifting in the temperature of the melt. It is known that the quantity of material used, especially in drug-excipient mixtures, could influence the peak shape and enthalpy. Thus, these minor changes in the melting endotherm of drug could be due to the mixing of drug and excipient, which lowered the purity of each component in the mixture, and this might not necessarily indicate potential incompatibility [17]. 

### 3.2. Particle Size, Poly Dispersity Index (PDI), and Zeta Potential of SLN

All the prepared samples were analyzed in order to determine their particle size distribution, zeta potential, and PDI values. The results are represented in Table 2. The particle size of all the formulations ranged from 167.8 nm to 271.76 nm, PDI from 0.230 to 0.441, and zeta potential from −18.5 to −28.1 mV. From the results obtained, formulations containing Dynasan-114 showed relatively lower particle sizes, but the PDI was higher and zeta potential was lower. Formulations containing Imwitor-900P showed lowest PDI, but higher particle sizes and lowest zeta potential where as formulations containing Dynasan-118 showed better size, PDI, and good zeta potential when compared to other formulations.

### 3.3. Entrapment Efficiency

Entrapment efficiency is an important parameter for characterizing solid lipid nanoparticles. All the formulations were analyzed for entrapment efficiency by HPLC, and the results are shown in Table 2. From the results obtained, all formulations showed good entrapment efficiency ranging from 82.38% to 92.06%. and formulation F1 and F2, F3 and F4 containing Dynasan-114 and Dynasan-118 showed relatively higher values compared to F5 and F6 containing Imwitor-900P.

### 3.4. Total Drug Content

All the formulations were analyzed for total drug content by HPLC, and the results are represented in Table 2. Formulations containing Dynasan-114 (F1 and F2) showed total drug content ranging from 9.1 mg to 9.5 mg and entrapment efficiency from 89.23% to 90.08%. Formulations containing Dynasan-118 (F3 and F4) showed total drug content ranging from 9.2 mg to 9.6 mg and entrapment efficiency from 91.56% to 92.06%. Formulations containing Imwitor-900P (F5 and F6) showed total drug content ranging from 9.0 mg to 9.4 mg and entrapment efficiency from 82.38% to 84.26%. In all these cases, the increase in lipid content could not improve the entrapment efficiency significantly.

### 3.5. *In Vitro* Drug Release

**Figure 2:**
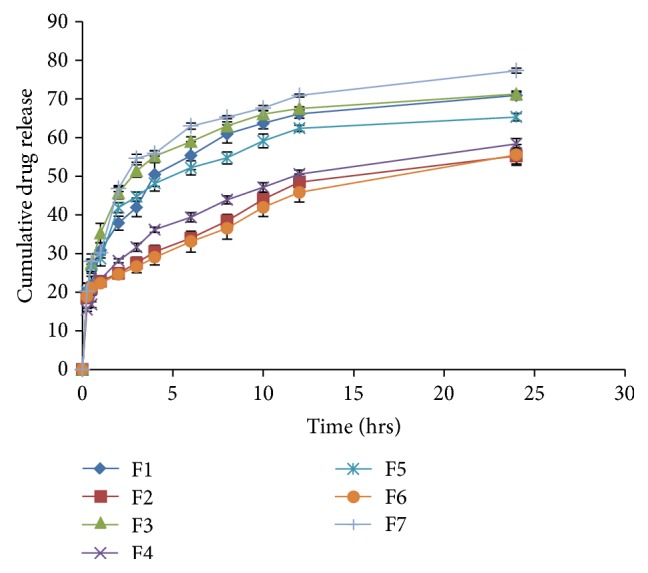
Cumulative % drug release from quetiapine fumarate SLNs in 0.1 N Hydrochloric acid.

Formulations containing Dynasan-114 (F1 and F2), Dynasan-118 (F3 and F4) and Imwitor-900P (F5 and F6) showed cumulative drug release ranging from 55.24% (F2) to 70.89% (F1), from 58.40% (F4) to 71.24% (F3) and from 55.55% (F6) to 65.32% (F5) respectively in 0.1 N Hydrochloric acid (Figure 2) during 24-hour period. Due to increased lipid content, the release was significantly retarded in all the prepared SLNs when compared in each set. Further, all SLNs released relatively slowly when compared to that of quetiapine suspension (F7). F3 formulation showed highest cumulative release (71.24%) among all the prepared SLNs, although not significantly different from F1 (70.89%). All the experiments were carried out in triplicate.

**Figure 3:**
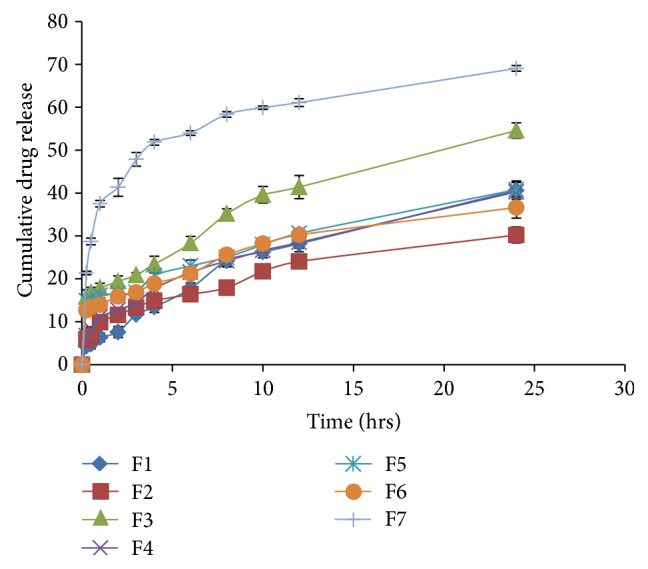
Cumulative % drug release from quetiapine fumarate SLNs in pH 6.8 phosphate buffer.

Formulations containing Dynasan-114 (F1 and F2) showed drug release ranging from 30.20% (F2) to 40.63% (F1) in 6.8 pH phosphate buffer. Formulations containing Dynasan-118 (F3 and F4) showed drug release ranging from 40.26% (F4) to 54.57% (F3) in 6.8 pH phosphate buffer (Figure 3).

Formulations containing Imwitor-900P (F5 and F6) showed drug release ranging from 36.63% (F6) to 40.80% (F5) in 6.8 pH phosphate buffer. When compared, the cumulative release in 6.8 pH phosphate buffer was less than that of 0.1 N HCl. Formulation (F3) containing Dynasan-118 showed maximum release of 54.47% in 6.8 pH phosphate buffer in 24 hours. In general, the *in vitro* release of drug from SLN is less in phosphate buffer pH 6.8 when compared to 0.1 N HCl. Quetiapine fumarate solubility is more in acid medium than in phosphate buffer pH 6.8. This increase in solubility is probably responsible for increase in the cumulative drug release in acid medium. Further, increased lipid content reduced the cumulative release which is in consistent with the earlier reports. The lipid content increases the packing density of lipid molecules in given space; as a result release is reduced. For further studies, based on the particle size, PDI, zeta potential, and *in vitro* release, F3 was considered as optimized formulation.

### 3.6. Physical Stability Studies of F3 Formulation during Storage

Quetiapine fumarate loaded solid lipid nanoparticles were stored at room temperature (25°C/60 ± 5%RH) and refrigerated temperature (4°C) for 60 days, and average size, zeta potential, poly dispersity index, and entrapment efficiency (EE) were determined. Stability studies were conducted for optimized formulation (F3) which showed better size, PDI, zeta potential, and EE. The number of samples estimated was in triplicate. The results are shown in Table 3. The statistical *t*-test was applied. Only size of SLNs was changed, but no significant difference was noticed in EE, PDI, and ZP of the SLNs during the storage period. Thus, the optimized SLN preparation was stable for two-months period at RT and at 4°C.

### 3.7. Bioavailability Study

**Figure 4:**
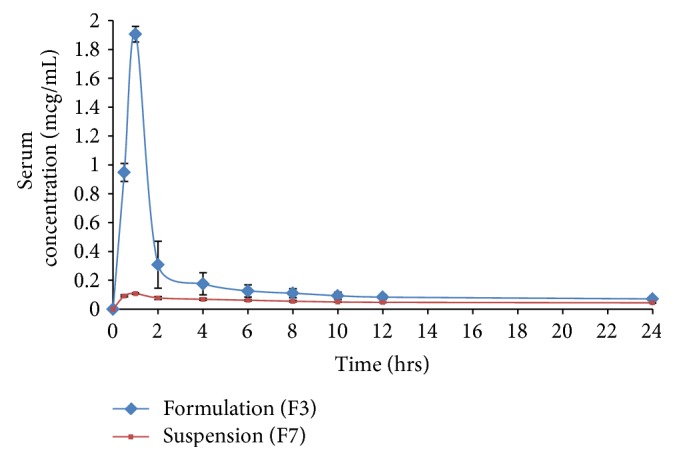
Serum concentration versus time profile of quetiapine fumarate upon oral administration of SLN preparation (F3) and suspension (F7) in rats.

The drug in the serum samples was estimated by using HPLC method [16]. Various pharmacokinetic parameters obtained for both suspension and SLN preparation following oral administration are given in Table 4. With SLN dispersion, the average peak plasma concentration of quetiapine fumarate is 1.902 ± 0.054 *μ*g/mL, whereas in the case of suspension, the peak plasma concentration is 0.107 ± 0.004 *μ*g/mL. The *t*_max_ is same for both suspension and SLN. The plasma concentration-time profiles of suspension and SLN formulation are shown in Figure 4. F3 profile is superior to that of suspension.

 From above results, it was found that *C*_max_ and AUC_0–24_ for suspension (F7) were lower than that of the optimized SLN formulation (F3). The pharmacokinetic parameters were subjected to statistical analysis using unpaired *t*-test with help of Graphpad Prism software version 5.0, 2007. Significant difference was noticed in *C*_max_, AUC, and *t*_1/2_ values for F3 when compared to the suspension (F7). Further, the relative bioavailability of the SLN formulation F3 was found to be 3.71 times that of suspension F7. The oral bioavailability of quetiapine fumarate from the formulation F3 was higher than that of suspension F7 formulation. This enhancement might be due to lymphatic transport of drug from SLN formulation and avoiding first-pass metabolism of drug.

## 4. Conclusion

Poorly bioavailable quetiapine fumarate was formulated as SLN using three different lipids after checking the compatibility by DSC studies. The SLN preparation with Dynasan 118 was optimized based on the particle size, PDI, zeta potential, entrapment efficiency, and drug release characteristics. During *in vivo* bioavailability studies 3.71 times of relative bioavailability improvement was found when compared to reference suspension. Thus, quetiapine fumarate when formulated as SLN could improve the oral bioavailability.

## Figures and Tables

**Table 1:** Composition of quetiapine fumarate loaded SLN formulations and suspension.

Formulation ingredient	Formulation code						
	F1	F2	F3	F4	F5	F6	F7
Organic phase							
Quetiapine fumarate (mg)	10	10	10	10	10	10	—
Dynasan-114 (mg)	100	200	—	—	—	—	—
Dynasan-118 (mg)	—	—	100	200	—	—	—
Imwitor 900P (mg)	—	—	—	—	100	200	—
Egg lecithin (mg)	100	100	100	100	100	100	—
Chloroform : methanol (1 : 1) (mL)	10	10	10	10	10	10	—
Aqueous phase							
Quetiapine fumarate (mg)	—	—	—	—	—	—	10
Sodium carboxymethyl cellulose (mg)	—	—	—	—	—	—	50
Poloxamer-188 (1.5% w/v) (mL)	10	10	10	10	10	10	—
Double distilled water (mL)	—	—	—	—	—	—	10

**Table 2:** Size, PDI, ZP, total drug content and EE of various formulations.

Formulation code	Size (nm) ± SD	PDI ± SD	ZP (mV) ± SD	Total drug content (mg) ± SD	EE (%) ± SD
F1	167.8 ± 11.87	0.441 ± 0.01	−23.9 ± 1.87	9.52 ± 0.01	89.23 ± 0.10
F2	194.23 ± 2.89	0.310 ± 0.01	−22.7 ± 1.78	9.11 ± 0.07	90.08 ± 0.24
F3	174.73 ± 4.47	0.305 ± 0.09	−28.1 ± 2.16	9.60 ± 0.02	91.56 ± 0.30
F4	207.16 ± 5.79	0.230 ± 0.01	−26.3 ± 2.06	9.23 ± 0.01	92.06 ± 0.09
F5	245.30 ± 4.92	0.241 ± 0.03	−19.9 ± 1.56	9.04 ± 0.00	82.38 ± 0.21
F6	271.76 ± 8.52	0.280 ± 0.08	−18.5 ± 1.45	9.40 ± 0.03	84.26 ± 0.25

**Table 3:** Physical parameters of the optimized formulation (F3) when stored at 25°C (RT) and (4°C) for a period of 2 months.

Day	At room temperature				At refrigerated temperature			
	Size (nm)∗	PDI	Zeta potential (mV)	EE (%)	Size (nm)∗	PDI	Zeta potential (mV)	EE (%)
1	174.73 ± 4.49	0.305 ± 0.09	−28.1 ± 2.16	91.5 ± 0.30	174.73 ± 4.7	0.305 ± 0.09	−28.1 ± 2.16	91.5 ± 0.30
30	205.56 ± 8.58	0.309 ± 0.14	−26.3 ± 2.06	87.2 ± 0.24	194.23 ± 2.8	0.310 ± 0.01	−26.1 ± 2.04	89.6 ± 0.42
60	210.9 ± 11.49	0.308 ± 0.06	−26.4 ± 2.08	82.1 ± 0.34	200.0 ± 8.5	0.311 ± 0.03	−26.6 ± 2.12	85.4 ± 0.18

The statistical comparison of data was done using unpaired *t*-test by GraphPad Prism software (version 5.0, 2007), and significance was calculated at *P* value of 0.05. ∗Significant difference was observed related to size of the SLN (*P* = 0.63). No significant difference was observed related to PDI, zeta potential, and EE during 1st, 30th and 60th day of storage.

**Table 4:** Consolidated table showing the pharmacokinetic parameters of quetiapine fumarate in rats—formulation with Dynasan-118 (F3) and suspension (F7)—a comparison (*n* = 6).

Pharmacokinetic parameters	Optimized formulation (F3)	Suspension (F7)
*C*_max_ (µg/mL)	1.902 ± 0.054 ∗	0.107 ± 0.004 ∗
*t*_max_ (h)	1 ± 0.00	1 ± 0.00
AUC_0–24_ (µg/mL) h	4.195 ± 0.623 ∗	1.129 ± 0.058 ∗
*t*_1/2_ (h)	11.662 ± 1.080 ∗	7.353 ± 0.935 ∗
MRT (h)	10.085 ± 0.500	11.270 ± 0.976

The statistical comparison of data was done using unpaired *t*-test by GraphPad Prism software (version 5.0, 2007), and significance was calculated at *P* value of 0.05. ∗Significant difference was observed between SLN formulation (F3) versus suspension (F7) in terms of *C*_max_, AUC, and *t*_1/2_. No significant difference was observed in terms of *t*_max_ and MRT.
